# Synthesis, Crystal Structure, Spectroscopic Properties and Potential Biological Activities of Salicylate‒Neocuproine Ternary Copper(II) Complexes

**DOI:** 10.3390/molecules20022115

**Published:** 2015-01-27

**Authors:** Lenka Kucková, Klaudia Jomová, Andrea Švorcová, Marián Valko, Peter Segľa, Ján Moncoľ, Jozef Kožíšek

**Affiliations:** 1 Department of Physical Chemistry, Faculty of Chemical and Food Technology, Slovak University of Technology, Radlinského 9, Bratislava SK-812 37, Slovak Republic; E-Mails: marian.valko@stuba.sk (M.V.); jozef.kozisek@stuba.sk (J.K.); 2 Department of Chemistry, Faculty of Natural Sciences, Constantine the Philosopher University, Trieda A. Hlinku 1, Nitra SK-949 74, Slovak Republic; E-Mails: kjomova@ukf.sk (K.J.); andrea.svorcova14@gmail.com (A.Š.); 3 Department of Inorganic Chemistry, Faculty of Chemical and Food Technology, Slovak University of Technology, Radlinského 9, Bratislava SK-812 37, Slovak Republic; E-Mails: peter.segla@stuba.sk (P.S.); jan.moncol@stuba.sk (J.M.)

**Keywords:** ternary copper complexes, biological activity, crystal structure

## Abstract

Mixed ligand copper(II) complexes containing derivatives of salicylic acid and heterocyclic ligands with nitrogen donor atoms have been the subject of various studies and reviews. In this paper, synthesis and characterization of the ternary copper(II) complexes of neocuproine (2,9-dimethyl-1,10-phenanthroline, Neo) and salicylate ligands (Sal) are reported. In addition, the crystal structures of ([Cu(H_2_O)(5-Cl-Sal)(Neo)] (**1**), [Cu(μ-Sal)(Neo)]_2_ (**2**), Cu_2_(μ-5-Cl-Sal)(5-Cl-HSal)_2_(Neo)_2_]·EtOH (**3**)) were determined. In order to compare structural and biological properties of the prepared complexes, spectroscopic and biological studies were performed. Results of X-ray diffraction show that prepared complexes form three types of crystal structures in a given system: monomeric, dimeric and dinuclear complex. The preliminary study on the DNA cleavage activity has shown that the complexes under study behave as the chemical nucleases in the presence of added hydrogen peroxide with slight differences in the activity (**1** > **2** > **3**). The complexes **1** and **2** exhibited nuclease activity itself indicating the interaction of complexes with the DNA. It has been proposed that the enhanced destructive effect of the complexes **1** and **2** on the DNA is a result of two possible mechanisms of action: (i) the conversion of closed circular DNA (form I) to the nicked DNA (form II) caused by the copper complex itself and (ii) damage of DNA by Reactive Oxygen Species (ROS)—products of the interaction of copper with hydrogen peroxide by means of Fenton reaction (hydroxyl radicals). Thus the biological activity of the prepared Cu(II) complexes containing derivatives of salicylic acid and phenanthroline molecules is substantiated by two independent mechanisms. While derivatives of salicylic acids in the coordination sphere of copper complexes are responsible for radical-scavenging activity (predominantly towards superoxide radical anion), the presence of chelating ligand 2,9-dimethyl-1,10-phenanthroline significantly enhances capability of Cu(II) complexes binding to DNA via intercalation.

## 1. Introduction

Despite advances in the development of antimicrobial agents, infectious diseases remain amongst the top five causes of mortality in the world [1]. One of the most crucial problems is growing drug resistance, especially the antibiotic resistance of pathogenic bacteria. Because such resistance can develop very quickly, there is a dire necessity to develop new antimicrobial compounds [2,3]. Although medicinal chemistry was almost exclusively based on organic compounds and natural products, during the past three decades metal complexes have gained a growing interest as pharmaceuticals due to their potential therapeutic applications [4].

Copper is one of essential elements required for normal human metabolism [5]. Copper(II) is known to play a significant role in biological systems and also as a pharmaceutical agent [6]. Its antibacterial properties have been known for thousands of years. Synthetic copper(II) complexes have been reported to act as a potential anticancer and cancer inhibiting agents and a number of copper complexes have been found to be active both *in vitro* and *in vivo* [7,8].

Nitrogen-containing ligands has found wide applications in chemotherapy and asymmetric catalysis. Among them, bipyridines and 1,10-phenanthrolines have been the most attractive due to their various functions [9]. The 1,10-phenanthroline (phen) and its derivatives exhibit antiviral, antifungal and antimycoplasmal activities [10]. It has been reported that stable bis-phen complexes inhibited DNA or RNA polymerase activities and induced strand scission of DNA in the presence of reducing agents (thiol or H_2_O_2_) [4,11,12,13,14,15]. DNA damage in the presence of Cu-phenanthrolines is attributed to the highly reactive hydroxyl radicals, OH· generated through the site-specific Fenton reaction [16].

Numerous biological studies have demonstrated that deoxyribonucleic acid (DNA) is the primary target molecule for most anticancer and antiviral therapies [17]. The question of the interaction and reaction of metal complexes containing multidentate aromatic ligands, especially *N*-containing ligands, with DNA, therefore, has long concentrated much attention in the development of new reagents for medicine and biotechnology [13]. This is due to their possible application as therapeutic agents and photochemical properties which make them potential probes of DNA structure and conformation. There are three distinct modes of non-covalent interaction of these metal complexes with DNA—intercalative association, DNA groove binding and electrostatic attraction—the nature of which is determined by the characteristics of the metal complexes [9].

Metal complexes with a variety of organic chelating ligand are also of current interest owing to their biological activities. These include anti-inflammatory and anticonvulsant properties [18], cytotoxicity and antiviral activity [19]. Among these, metal complexes of carboxylates have been intensively studied because the carboxylate group can be coordinated to the metal ion in various ways. These ligands can bind as neutral molecular ligands or as anionic ligands in monodentate, bidentate or bridging manner [20]. Particularly, salicylate copper complexes are of growing interest from both structural and biological point of view. Salicylic acid itself and its healing properties have been known for centuries [21]. It is well known that salicylic acid and its derivatives are non-steroidal anti-inflammatory, anti-pyretic and analgesic drugs [22]. Copper complexes of anti-inflammatory drugs have been shown to be more potent and desirable drugs compared to ligands themselves [23]. Furthermore, it is reported that some of their pharmacological activities are enhanced in the presence of ancillary nitrogen donor ligands [24]. Copper(II) has been shown to act in a synergistic manner with the salicylic acid derivatives [25]. From the coordination chemistry point of view, salicylic acid is a versatile ligand as it has two donor centers in a ligand geometry facilitating metal bridging or chelation for medium or large size cation. In addition, the hydroxyl group in the salicylate ligand can participate in intra- and intermolecular hydrogen bonds [26].

In the literature, there are few reports concerning ternary complexes of Cu(II) with salicylates and substituted phenanthrolines [27,28,29]. Among them, bis(diisopropylsalicylato)-(2,9-dimethyl-1,10-phenanthroline)copper(II) complex seems to be the most potent. It exhibits cytotoxicity comparable with the anticancer drug, cisplatin [PtCl_2_(NH_3_)_2_] [23].

In this paper, the synthesis, crystal structure, as well as spectral and preliminary biological activity of the compounds aqua(5-chloro-2-oxidobenzoato-κ^2^*O*,*O'*)(2,9-dimethyl-1,10-phenanthroline-κ^2^*N*,*N'*)copper(II) [Cu(H_2_O)(5-Cl-Sal)(Neo)] (**1**), bis[(μ-2-oxidobenzoato-κ*O*:κ^2^*O*,*O'*)-(2,9-dimethyl-1,10-phenantroline-κ^2^*N*,*N'*)copper(II)] [Cu(μ-Sal)(Neo)]_2_ (**2**) and (μ-5-chloro-2-oxidobenzoato-κ^2^*O*,*O'*:κ^2^*O'*,*O''*)-bis[(5-chloro-2-hydroxy-benzoato-κ^2^*O*,*O'*)(2,9-dimethyl-1,10-phenantroline-κ^2^*N*,*N'*) copper(II)] ethanol solvate [Cu_2_(μ-5-Cl-Sal)(5-Cl-HSal)_2_(Neo)_2_]·EtOH (**3**) (where H_2_Sal is salicylic acid, Neo is neocuproine, *i.e*., 2,9-dimethyl-1,10-phenanthroline) are reported.

## 2. Results and Discussion

### 2.1. Crystal Structures

#### 2.1.1. [Cu(H_2_O)(5-Cl-Sal)(Neo)] (**1**)

**Figure 1:**
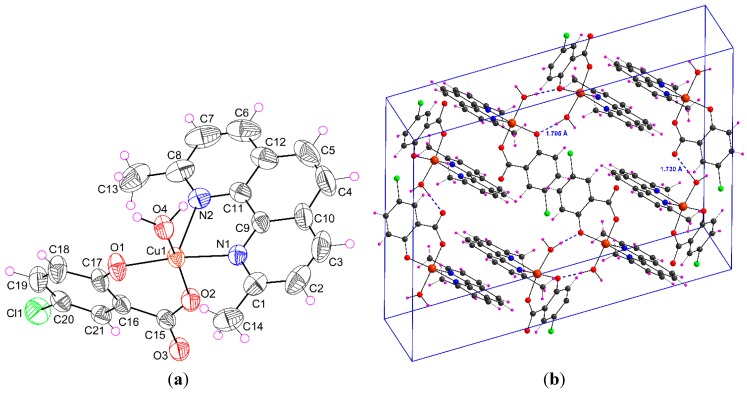
(**a**) Perspective view of complex **1**, with the atom numbering scheme. Thermal ellipsoids are drawn at the 50% probability level; (**b**) View of the packing of **1** in the unit cell with hydrogen bonds (dashed blue lines).

Complex **1** is monomeric and the central atom is coordinated by five donor atoms. The molecular structure of this ternary mononuclear complex is depicted in Figure 1a and selected bond lengths and angles are given in Table 1. The coordination polyhedron around the central copper atom has the form of a deformed square pyramid. The basal plane is formed by one nitrogen atom of Neo [Cu1–N1 = 2.023(2) Å], phenolate [Cu1–O1 = 1.899(2)] and carboxylate [Cu1–O2 = 1.924(2) Å] oxygen atoms of anion ligand (5-Cl-Sal^2−^) and the oxygen atom of solvent that is water in this case [Cu1–O4 = 1.973(2) Å]. The coordination of copper is completed by second nitrogen atom N2 of Neo that is in axial position [Cu1–N2 = 2.295(2) Å]. For this point of view, a geometry parameter *τ*, which is defined *τ* = (*β* − *α*)/60, applicable to 5-coordinate structures within the structural continuum between trigonal bipyramidal and tetragonal or rectangular pyramidal is defined. For a perfect tetragonal pyramid *τ* is zero, and for a perfect trigonal-bipyramidal geometry *τ* becomes 1.0 [30]. In the compound **1**, the largest angles within the four atoms O1, O2, O4, N1, are *β* = 168.40(8)° for O1–Cu1–N1, and *α* = 163.88(9)° for O2–Cu1–O4. Thus, *τ* is (168.40 − 163.88)/60 = 0.076, indicating a 92% tetragonal pyramidal geometry.

**Table 1:** Selected bond lengths (Å) and angles (°) for compounds **1**, **2** and **3**.

1			
Cu1–N1	2.023(2)	O1–Cu1–O2	93.34(7)
Cu1–N2	2.295(2)	O2–Cu1–N1	88.45(7)
Cu1–O1	1.899(2)	N1–Cu1–N2	77.57(8)
Cu1–O2	1.924(2)	N1–Cu1–O4	86.97(7)
Cu1–O4	1.973(2)	O4–Cu1–O1	88.19(7)
**2**			
Cu1–N1	2.000(3)	O2–Cu1–N1	90.80(12)
Cu1–N2	2.257(3)	O2–Cu1–N2	99.41(11)
Cu1–O1	1.991(2)	N1–Cu1–N2	79.62(13)
Cu1–O2	1.909(3)	O1–Cu1–O1 ^i^	76.37(12)
Cu1–O1 ^i^	1.919(2)	Cu1–O1 ^i^–Cu1 ^i^	100.51(11)
Cu1–Cu1 ^i^	3.007(1)	O2–Cu1–O1	152.18(11)
**3**			
Cu1–N1	2.004(3)	O1–Cu1–O2	90.58(11)
Cu1–N2	2.016(3)	O1–Cu1–N1	145.46(12)
Cu2–N3	2.023(4)	O2–Cu1–N1	99.86(13)
Cu2–N4	2.301(4)	N1–Cu1–N2	83.30(13)
Cu1–O1	1.934(2)	O6–Cu2–O5	92.60(11)
Cu1–O2	1.967(3)	O7–Cu2–N4	81.46(13)
Cu1–O3	2.471(3)	N3–Cu2–N4	77.68(15)
Cu1–O5	2.744(2)	O2–Cu1–N2	147.57(12)
Cu2–O5	1.949(2)	O1–Cu1–N2	105.08(12)
Cu2–O6	1.896(3)	O7–Cu2–N4	81.46(13)
Cu2–O7	1.966(3)	O7–Cu2–N3	89.59(12)
Cu2–O8	3.167(2)	O6–Cu2–N4	111.36(11)

Note: Symmetry code: (i) −*x*, *y*, 1/2−*z.*

In the Cambridge Structural Database there is one crystal structure similar to Neo, refcode DAZNEY01, with the corresponding distances: Cu–N 2.033 Å and 2.280 Å for Cu–O bond, and *τ* parameter is 0.117 [31]. The main difference in analogous crystal structures of 1,10-phenanthroline with the chromophore [CuN_2_O_3_] (HAXMOK, MOSZAW, TITQX) is that both nitrogen ligands are coordinated in equatorial plane. Interatomic distances for Cu–O and Cu–N in equatorial plane are in the interval of 1.880–1.904 Å and 2.001–2.033 Å, as well. For the apical oxygen atom the distances in these three crystal structures are 2.582, 2.329 and 2.349 Å, as well [29,32,33].

**Figure 2:**
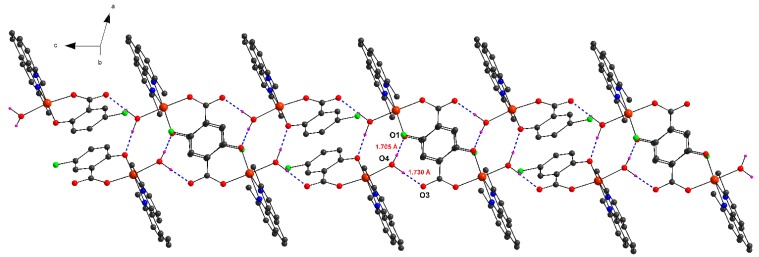
1D chain formed by hydrogen bonds in complex **1**.

Intermolecular hydrogen bonds (Table 2) and π-π aromatic interactions have a key role in stabilizing complex **1** in the solid state. A strong O–H···O interactions (Figure 1b, 2) were observed between hydroxyl oxygen (O1) and water –OH group (<O–H···O 173.6°) and/or carboxylate oxygen (O3) and water –OH group (<O–H···O 174.8°). These interactions form 1D chains in the crystal structure as shown in Figure 2. The monomeric complex is further stabilized by π-π aromatic stacking interactions between neocuproine ligands from neighboring molecules (interplanar distances d(C–C) = 3.857–3.956 Å).

**Table 2:** Observed hydrogen bonds for studied complexes **1** and **2** (Å, °).

*D*–H···*A*	*D*–H	H···*A*	*D···A*	*D*–H···*A*
**1**				
O4–H4W···O3 ^i^	0.950(5)	1.730(6)	2.678(2)	174.83(3)
O4–H5W···O1 ^ii^	0.954(4)	1.705(6)	2.655(2)	173.56(2)
**2**				
C7–H7A···O2 ^iii^	0.950(4)	2.641(4)	3.413(5)	138.70(2)

Note: Symmetry codes: (i) *x*, −*y*+1, *z*+1/2; (ii) −*x*, *y*, −*z*+1/2; (iii) *x*, *y*+1, *z*.

#### 2.1.2. [Cu(μ-Sal)(Neo)]_2_ (**2**)

**Figure 3:**
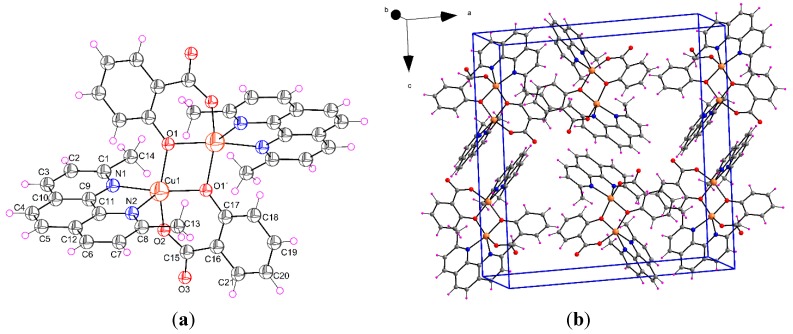
(**a**) Perspective view of complex **2**, with the atom numbering scheme. Thermal ellipsoids are drawn at the 50% probability level (symmetry code i: −*x*, *y*, 1/2−*z*); (**b**) View of the packing of **2** in the unit cell.

The crystal structure of complex **2** is represented in Figure 3. Single crystal X-ray diffraction analysis reveals that a dimeric complex **2** contains [Cu(II)(Neo)(Sal)] moiety. Each pair of moieties are aligned as a centrosymmetric dimeric unit (Cu–Cu ^i^ distance: 3.007(1) Å, symmetry code i: −*x*, *y*, 1/2−*z*). The coordination geometry of Cu(II) ions in the moieties is five-coordinate, deformed square pyramidal. The basal plane is formed by one nitrogen atom from a neutral Neo ligand [Cu1–N1 = 2.000(3) Å], phenolate [Cu1–O1 ^i^ = 1.919(2) Å] and carboxylate [Cu1–O2 = 1.909(3) Å] oxygen atoms of the Sal^2−^ (salicylic acid with two deprotonated oxygen atoms) ligand and an oxygen atom of a phenolate group of the Sal^2−^ ligand from another [Cu(II)(Neo)(Sal)] moiety [Cu1–O1 = 1.991(2) Å]. The coordination of copper is completed by one apical copper-nitrogen atom [Cu1–N2 = 2.257(3) Å] from Neo ligand. Selected bond parameters (distances and angles) of **2** are shown in Table 1. It is apparent (Figure 3) that nitrogen atoms are not bonded in the same way, one nitrogen atom (N1) is bonded in equatorial position, whereas another one (N2) occupies the axial position. Copper atoms in dimeric unit do not interact with each other because the distance Cu–Cu ^i^ is 3.007(1) Å. The coordination geometry of the Cu^2+^ ions in dimer are very similar to those in monomers, with the exception that phenolate O atom, that forms the bridge between the Cu^2+^ ions, occupies the fifth coordination place instead of the O atom from solvent molecule. In this complex, the phenolate atom of Sal^2−^ is both bridging and chelating. Interestingly, this type of coordination was reported recently by Moncol *et al*., (2012) [29]. In the compound **2**, the largest angles within the four atoms O1, O1 ^i^, Cu1, Cu1 ^i^, are *β* = 168.95(12)° for O1 ^i^–Cu1–N1, and *α* = 152.18(11)° for O1–Cu1–O2. Thus, *τ* is 0.280, indicating a 72% rectangular pyramidal geometry.

In the Cambridge Structural Database there are two crystal structures similar to phenanthroline, refcode LEHFEK and KURQIY [34,35]. The corresponding distances between two Cu atoms in these compounds are 3.138(2) Å and 3.312(2) Å, respectively. Interatomic distances for Cu–O and Cu–N in equatorial plane are in the interval of 1.882–1.906 Å and 1.999–2.028 Å, as well. The distances of the corresponding apical oxygen atom in these two crystal structures are 2.569 and 2.389 Å, as well. Similarly to monomeric complex, the main difference between analogous dimeric structures is that both nitrogen atoms of phenanthroline are coordinated in equatorial plane.

In the solid state, the crystal structure of **2** is stabilized by strong C–H…O interactions (Table 2). In addition to these relatively strong interactions, π-π aromatic stacking interactions between neocuproine ligands from neighboring molecules (interplanar distances d(C–C) = 3.449 Å) are observed. As a result of these secondary interactions a 3D supramolecular assembly is formed in the solid state.

#### 2.1.3. [Cu_2_(μ-5-Cl-Sal)(5-Cl-HSal)_2_(Neo)_2_]·EtOH (**3**)

**Figure 4:**
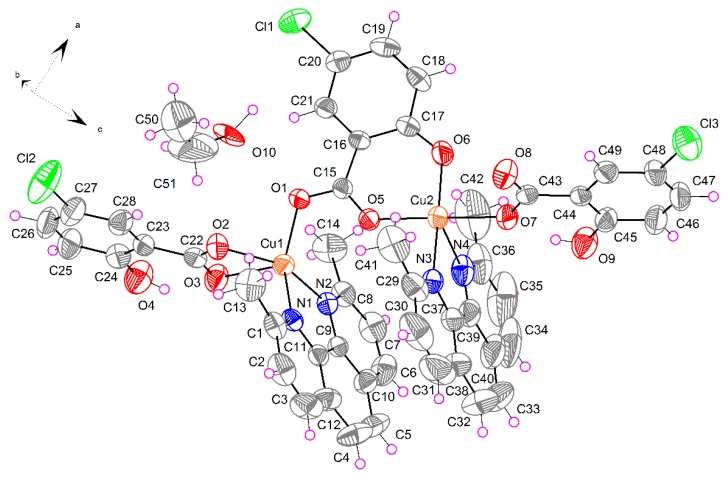
Perspective view of complex **3**, with the atom numbering scheme. Thermal ellipsoids are drawn at the 50% probability level.

The crystal structure of binuclear complex **3** is shown in Figure 4. The ratio of Cu:5-Cl-Sal:Neo in complex **3** is 2:3:2, where one derivative of salicylic acid has two deprotonated oxygen atoms (5-Cl-Sal^2−^), while two salicylic acids have one deprotonated oxygen atom (5-Cl-HSal^−^). The coordination environment of one copper (Cu2) is deformed square pyramidal, whereas the coordination polyhedron of second copper (Cu1) has the form of deformed square bipyramid. The tetragonal plane of Cu1 is built up by a pair of nitrogen atoms from Neo [Cu1–N1 = 2.004(3) and Cu1–N2 = 2.016(3) Å] and a pair of carboxylate oxygen atoms from 5-Cl-HSal^−^ possessing one deprotonated oxygen [Cu1–O2 = 1.967(3) Å] and from bridging anion ligand (5-Cl-Sal^2−^) [Cu1–O1 = 1.934(2)]. The axial positions are occupied by two carboxyl oxygen atoms from the same anion ligands [Cu1–O3 = 2.471(3) and Cu1–O5 = 2.744(2) Å]. The basal plane of Cu2 is formed by one nitrogen atom from a neutral Neo ligand [Cu1–N3 = 2.023(4) Å], phenolate and carboxylate oxygen atoms of bridging 5-Cl-Sal^2−^ ligand and one oxygen atom of another 5-Cl-HSal^−^ ligand [Cu1-O_eq_ are in the range 1.896(3)–1.966(3) Å]. The coordination of copper Cu2 is completed by one apical copper-nitrogen atom distance [Cu2–N4 = 2.301(4) Å] from Neo ligand.

In compound **3**, for atom Cu1, the largest angles within the four atoms O1, O3, O5, N1, are *β* = 146.65(11)° for O5–Cu1–O3, and *α* = 145. 46(12)° for N1–Cu1–O1. Thus, *τ* is (146.65 − 145.46)/60 = 0.020. For atom Cu2, the largest angles within the four atoms O5, O6, O7, N3, are *β* = 176.59(13)° for O5–Cu2–O7, and *α* = 170. 75(14)° for N3–Cu2–O6. Thus, *τ* is (176.59 − 170.75)/60 = 0.097.

**Figure 5:**
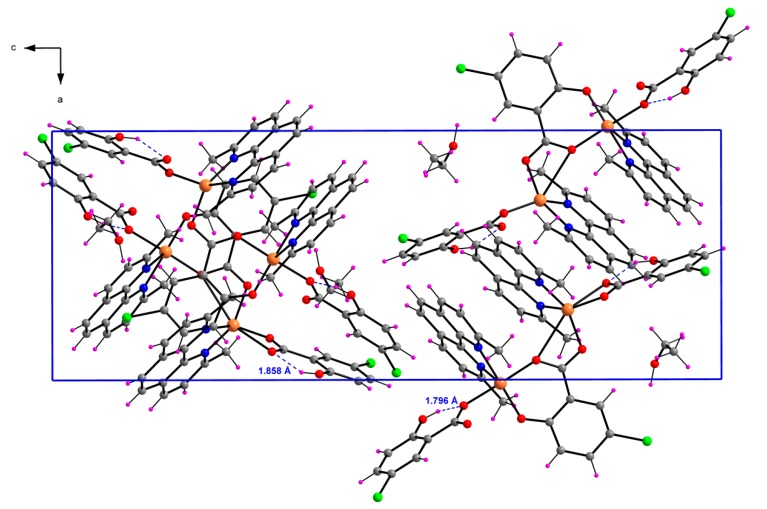
View of the packing of **3** in the unit cell with intramolecular hydrogen bonds (dashed blue lines).

The dominant feature of complex **3** is the strong intramolecular hydrogen bond between hydroxyl OH group and carboxylate oxygen atom (Figure 5). Additional C–H…O interactions and π-π aromatic stacking interactions are observed. These interactions are summarized in Table 3. As a result of these secondary interactions a 3D supramolecular assembly is formed in the solid state.

**Table 3:** Observed hydrogen bonds for complexes **3** (Å, °).

*D*–H···*A*	*D*–H	H···*A*	*D*···*A*	*D*–H···*A*
O4–H4H···O3	0.820(4)	1.858(9)	2.583(4)	146.7(2)
O9–H9H···O7	0.820(4)	1.796(9)	2.523(4)	146.8(9)
O10–H10W···O8 ^i^	0.970(4)	1.830(9)	2.762(6)	160.3(2)
C2–H2A···O3 ^ii^	0.950(5)	2.524(8)	3.433(5)	160.2(2)
C13–H13A···O2	0.980(8)	2.331(7)	3.053(5)	129.8(2)
C14–H14A···O1	0.980(7)	2.553(9)	3.164(5)	120.5(2)
C41–H41A···O5	0.980(7)	2.581(9)	3.245(6)	125.1(2)
C47–H47A···O10 ^iii^	0.950(5)	2.527(8)	3.462(5)	168.3(2)

Note: Symmetry codes: (i) −*x*+2*, y*−1/2, *−z*+1/2; (ii)−*x*+1*, y*+1/2, *−z*+1/2; (iii)* −x*+5/2, *−y*+1, *z*+1/2.

### 2.2. Spectroscopic Data

#### 2.2.1. IR Data

**Figure 6:**
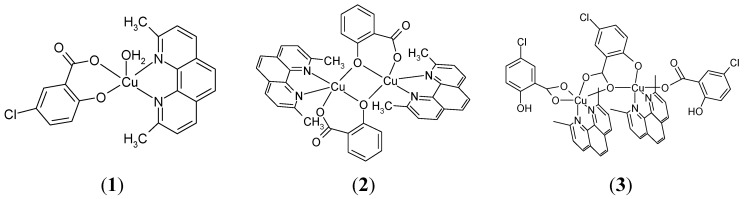
Proposed structures of the discussed complexes.

The proposed structures of studied complexes are depicted in Figure 6. The vibrational spectra of salicylic acid, 5-chlorosalicylic acid, sodium salicylate and copper(II) salicylate adducts with neocuproine are given in Table 4.

**Table 4:** IR data (cm^−1^) for H_2_Sal, 5-ClH_2_Sal, sodium salicylate (NaHSal) and copper(II) salicylate adducts with Neo.

	Ph–OH or Ph–O^−^ Group			COOH or COO^−^ Group					
	*ν*(O–H)	*ν*(C–O)	Type of Coordination	*ν*(C=O)	*ν*_as_(COO^−^)	*ν*_s_(COO^−^)	*ν*(C–O)	∆ ^b^	Type of Coordination
H_2_Sal	3230m	1291vs		1653s, br			1235s, br		
5-ClH_2_Sal	3235m	1288vs		1659s, br			1219s1230sh		
NaHSal	3219w	1284vs, br	non-coordinated		1578vs ^a^	1373vs		205	ionic
(**1**)		1307vs	unidentate		1598vs ^a^	1358s		240	unidentate
(**2**)		1301s	bridging		1593s ^a^	1358vs		235	unidentate
(**3**)	3218w	1325s1290m	unidentatenon-coordinated		1618vs1600vs1588vs ^a^	1364m 1406vs 1426vs		254194162	unidentateasym. chelatingbridging

Notes: vs, very strong; s, strong; m, medium; w, weak; sh, shoulder; br, broad. ^a^ Mixed bands or some of the observed bands also belong to another vibrational mode. ^b^ ∆ = *ν*_as_(COO^−^) − *ν*_s_(COO^−^).

All typical features of the IR spectra are clearly compatible with the structural characteristics of the complexes under study. Due to the complexity of the spectra, we will analyze the vibrational modes of the main groups (phenolic, carboxylic, aromatic ring) separately, as much as possible.

##### Phenolic Group Vibrations

In the spectra of the free acids H_2_Sal, 5-ClH_2_Sal as well as sodium salicylate NaHSal (Table 4) we observe bands originating from the stretching vibrational mode of the phenolic Ph–OH group above 3200 cm^−1^ assigned to *ν*(O–H) and under 1300 cm^−1^ assigned to *ν*(C–O). The position of bands which correspond to *ν*(C–O) stretching vibration of Ph–OH group in free acids and sodium salicylate are shifted for complexes **1**, **2** and **3** by about 20 cm^−1^ to higher wave numbers. Based on this shift and absence of band assigned to *ν*(O–H) stretching vibration of phenolic group [36,37], we can assume the deprotonated Ph-O^–^ group is present in the complexes under study. The crystal structures of studied complexes have confirmed these modes of coordination: phenolate groups of salicylate anions are monodentate coordinated through phenolate oxygen atom in complexes **1** and **3** or bidentate bridging in complex **2** to Cu(II) atoms (see crystal structures of complexes). Similarly, it has been shown in the literature that the band for phenol at 1240 shifts to 1270 cm^−1^ when the phenolic oxygen loses its hydrogen [38]. Finally, in the spectrum of complex **3** we observe the band at 3218 as well as at 1290 cm^−1^, which indicates also the presence of non-deprotonated as well as non-coordinated PhOH group in complex **3**.

##### Carboxyl Group and Neocuproine Vibrations 

The strong bands assigned to asymmetric and symmetric stretching vibration for complexes **1**–**3**, which contain salicylate anions are in the regions at about 1600 and below 1400 cm^−1^, respectively. The differences (∆) between the asymmetric stretch *ν*_as_(COO^−^) and symmetric stretch *ν_s_*(COO^−^) vibrations give information on the carboxylate bonding mode for the complexes after comparison with ∆ values for compounds with ionic carboxylic groups [39]. For sodium salicylate the ∆ value (Table 4) is 205 cm^−1^. The greater ∆ values for complexes **1** and **2** suggest unidentate coordination of the carboxyl group of 5-Cl-Sal^2−^ and Sal^2−^, respectively. In the IR spectrum complex **3** we observe considerable splitting of bands assigned to asymmetric and symmetric stretching vibration of the carboxyl group, which is due to various modes of coordination. The ∆ values for complex **3** are typical for monodentate, chelating asymmetrical as well as bridging coordination of the carboxyl group. The suggested coordination of phenolate group as well as carboxylate group salicylate anions for complexes under study is in agreement with the structures determined by X-ray analysis.

The intense bands at about 1590 cm^−1^ (assigned to the asymmetric stretch *ν*_as_(COO^−^)) are often overlapped with the stretching vibration of C=N (aromatic bond) of the phenanthroline ring are present in all complexes. The medium to low intensity bands at 534–560 and 412–431 cm^−1^ are attributed to the coordination bonds (Cu–O and Cu–N, respectively).

#### 2.2.2. Electronic Data

The solid state electronic spectra of all three copper(II) complexes under study exhibit a symmetrical broad ligand field band (band I) with a maximum at 14,700 (complex **1**), 13,300 (complex **2**) and 12,700 cm^−^^1^ (complex **3**). For complex **1**, there is also a charge transfer band at about 25,000 cm^−1^ (band II). This type of d–d spectra for all complexes under study is typical for pentacoordinated square-based pyramidal or tetragonally distorted octahedral copper(II) complexes [40]. There is certainly a general trend, that as distortion proceeds from regular square pyramidal to regular trigonal bipyramidal (to say, *τ* = 0.076 for complex **1**, *τ* = 0.280 for complex **2** and *τ* = 0.020 or 0.097 respectively for complex **3**), maximum intensity of the absorption band I shifts to lower energy.

#### 2.2.3. EPR Spectroscopy

**Figure 7:**
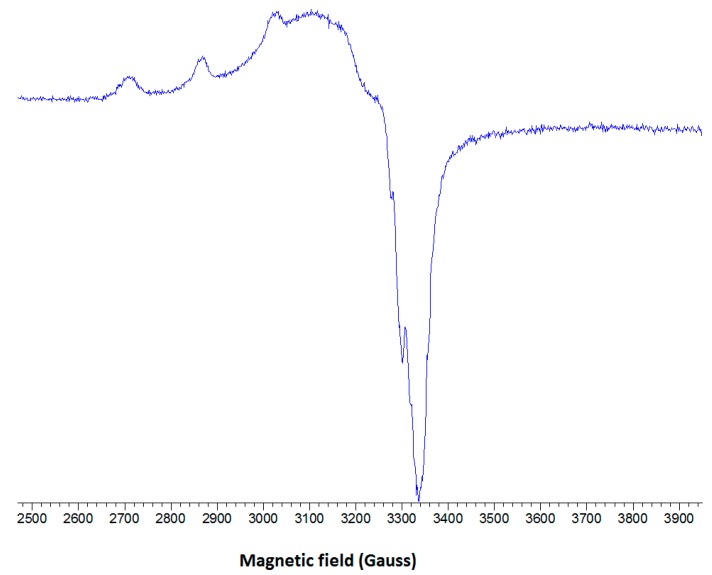
The low temperature EPR spectrum of complex 1 in frozen DMSO solution at 77 K.

The low-temperature X-band EPR spectrum of complex **1** is shown in Figure 7. The spectrum shows three well-resolved low-field parallel lines with a hyperfine splitting (A_||_) of 153 Gauss and g_||_ = 2.316. The fourth parallel line is obscured by the much stronger perpendicular portion of the spectrum which exhibits signs of hyperfine splitting (~20 Gauss).

Detailed correlations between g_||_ and A_||_ have been summarized for various copper(II) environments [40]. It has been well established, that tetrahedral distortions of a planar CuX_4_ moiety markedly reduce A_||_ while simultaneously increasing g_||_. The tetrahedral distortion arising from the dependence of g_||_ on the dihedral angle led to the introduction of the quotient g_||_/A_||_ as a convenient measure of the degree of tetrahedral distortion [41,42]. The EPR data for complex **1** give a value of 151 cm which suggests a mild tetrahedral distortion around the copper center in the basal plane. This conclusion is in agreement with the X-ray data discussed above and points out that the solvent DMSO has a moderate influence on the coordination geometry of complex **1**.

**Figure 8:**
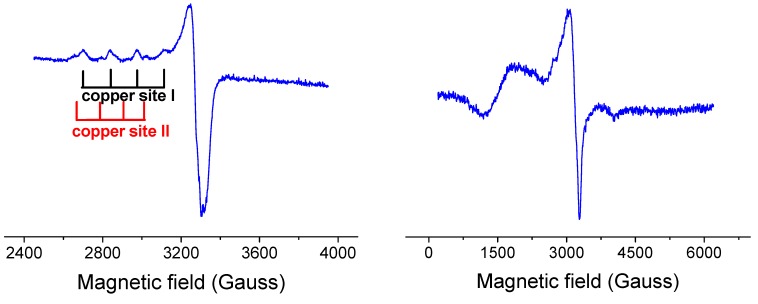
The low temperature EPR spectrum of dimeric complex **3** in frozen DMSO solution (left). EPR spectrum of complex **3** in solid state at 77 K showing typical features of dimeric species. Spectral parameters: g_﬩_ = 1980, g_ǁ_ = 2290, D = 1180.10^−4^ cm^−1^, E = 300.10^−4^ cm^−1^ (right).

The X-ray diffraction analysis for **2** and **3** revealed dimeric structures. However, the EPR spectra of both complexes **2** (spectrum not shown) and **3** (Figure 8) in frozen DMSO solutions are typical for monomeric species with no features of dimeric species, suggesting the breaking of the dimers into monomeric species. This result is not very surprising since DMSO is a solvent of high solvation ability (DMSO has one of the highest Gutmann donor number *D*_s_ = 27 of all solvents) which can strongly coordinate to the metal ions and is even able to compete with various types of ligands in the first coordination sphere of the Cu(II) ion.

**Figure 9:**
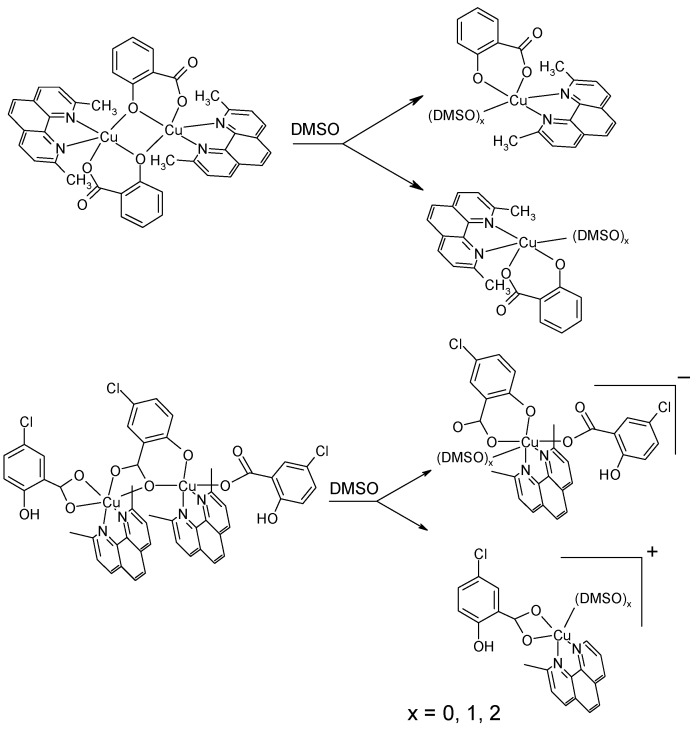
Schematic drawing of the decomposition of complexes **2** and **3** in DMSO.

For illustration, the EPR spectrum of complex **3** in frozen DMSO solution is shown in Figure 8. It shows typical monomeric Cu(II) EPR signal with well resolved three of four parallel hyperfine lines (copper site I) suggesting a marked tetrahedral distortion around copper ion (quotient g_||_/A_||_ being 175 cm). In addition, low abundant copper(II) species (copper site II) with different coordination geometry were detected. Thus, in case of complex **3** in DMSO we may expect an equilibrium between two monomeric coordination environments around copper(II) ion. We suggest that complex **3** in DMSO breaks down into monomeric cation Cu(II) complex and monomeric anion Cu(II) complex (see Figure 9). A more detailed description of the newly formed structures in solution is impossible since the superhyperfine EPR structure originating from magnetically active nitrogens has not been resolved. The only structural information about monomeric complexes formed in DMSO can be derived from the sufficiently resolved EPR spectra. However, the EPR spectrum of **3** in DMSO provided only partial resolution so the detailed description of coordination environment around copper ion was not possible to make.

Since the dimeric core of complex **2** is a symmetric one, we may expect the cleavage of complex **2** into two structurally identical molecular monomeric Cu(II) complexes (see Figure 9). Thus based on the EPR spectra of dimeric copper complexes in DMSO solution we may expect that complex **2** exists in equilibrium of two molecular Cu(II) complexes and complex **3** in equilibrium of cation and anion Cu(II) complexes. Formation of Cu(II) cation/anion complexes for **3** may have an important impact on their interaction with plasmid DNA (see below). A simple scheme of the decomposition of both complexes **2** and **3** in DMSO is outlined in the Figure 9.

### 2.3. DNA Cleavage Activity

The ability of the prepared complexes **1**–**3** to induce a cleavage of DNA (nuclease-like activity) was investigated by the gel electrophoresis following reaction with pBSK+ plasmid. The method of gel electrophoresis allows distinguishing the DNA conformational states based on their relative mobility in the gel. Plasmid DNA, naturally appearing as a covalently closed circular form (I, supercoiled), can be cleaved on one strand leading to the formation of a slower moving relaxed/nicked conformation (Form II). The cleavage of both strands results in the formation of a linear DNA (Form III) that migrates in between the positions of forms I and II.

**Figure 10:**
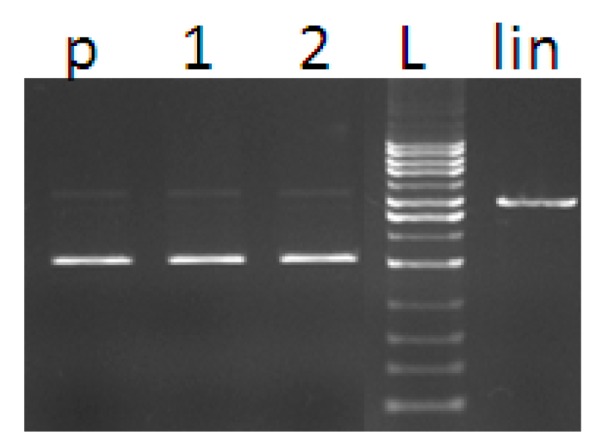
Agarose gel electrophoresis (0.8%) of pBSK+. Lanes represent: (p) control plasmid, (1) plasmid + H_2_O_2_, (2) plasmid + DMSO, (L) 1kb DNA ladder, (lin) plasmid linearised with Eco RI restrictase.

**Figure 11:**
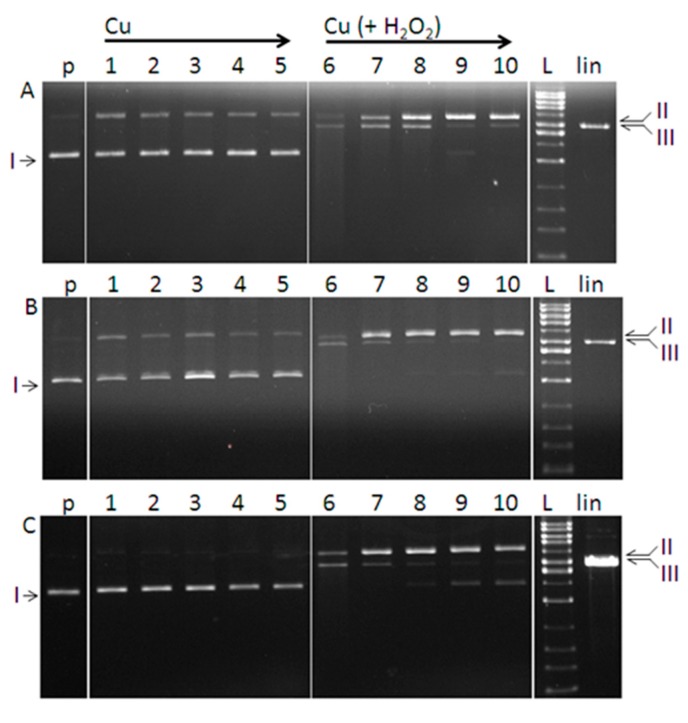
Agarose gel electrophoresis (0.8%) of pBSK+ plasmid cleavage by Cu(II) complexes. All reactions were in a final volume of 20 μL, for 1 h at 37 °C. Lanes represent: (p) control plasmid, (1–5) plasmid + copper complex (5, 10, 25, 40, 50 µM); (6–10) plasmid + copper complex (5, 10, 25, 40, 50 µM) each with 50 µM H_2_O_2_; (L) 1kb DNA ladder, (lin) plasmid linearised with Eco RI restrictase. (**A**–**C**) refer to complex **1**, **2** and **3**, respectively.

Plasmid DNA was exposed to different concentrations of each complex (5–50 µM). The complexes dissolved in DMSO were studied alone and together with hydrogen peroxide at fixed concentration (50 µM). Figure 10 shows that cleavage does not occur when hydrogen peroxide and DMSO are added separately into the DNA solution. Activities of the complexes are shown in Figure 10. In the absence of added hydrogen peroxide, complexes **1** and **2** show comparable ability to break plasmid DNA (form I) into relaxed conformation (Figure 11A,B, Line 1–5). In contrast, complex **3** shows no ability to interact with closed circular DNA within the concentration range tested (Figure 11C, Line 1–5). The nuclease activity of complexes **1** and **2** was probably due to the intercalation of neocuproine moiety of the complex into the DNA molecule. We note that the method of gel electrophoresis is a suitable (though indirect) method for investigation of interaction of DNA with complex compounds. The occurrence of a certain amount of nicked conformation state of the plasmid DNA in the presence of copper complex compound without hydrogen peroxide (observed in our experiments for complexes **1** and **2**) can be considered as evidence of an interaction of the copper complex with the DNA. Our assumption is supported by the findings of Maheswari *et al.* [43] who observed nuclease activity of copper complex compound as a result of the intercalation of the planar ligand into the DNA base pairs and a direct cleavage of the strands, but without ROS production. We can exclude a weak van der Waals interaction which cannot lead to the cleavage of the covalent bond in the DNA strand. The higher nuclease activity of complexes **1**, **2** in the presence of hydrogen peroxide compared to the activity of complex **3** supports our assumption and the higher activity is justified as a contribution of both possible mechanisms of action of the complexes.

The cleavage activity of all the complexes was observed in the presence of hydrogen peroxide (Figure 11A–C, Line 6–10), indicating the involvement of hydrogen peroxide in the reaction with the metal ion followed by DNA degradation. The results are consistent with the number of previous studies conducted with both cupric and cuprous complexes [42,43,44,45,46] which confirmed the nuclease-like activity supported by the presence of agents, such as hydrogen peroxide or ascorbic acid. The cleavage of DNA in the presence of added hydrogen peroxide as a co-reactant supports the theory of oxidative damage of DNA that is believed to contribute to the cytotoxic potency of the complexes [43].

Hydrogen peroxide primarily acts as an oxidizing agent in the Fenton reaction (Cu(I) + H_2_O_2_ → Cu(II) + OH^−^ + ^•^OH), in which cuprous ions interact with hydrogen peroxide. However, in case of coordination compounds containing copper in oxidation state 2+ (or alternatively iron in oxidation state 3+) these compounds are able to promote hydroxyl radical formation only in the presence of reducing agents. Examples of reducing agents in biological systems include glutathione, ascorbic acid, and other molecules. It is rational to assume in the case of complexes **1**, **2** and **3** coordinated ligands bearing nitrogen donor atoms possess capacity to partially reduce Cu^2+^ to Cu^+^ ions [47,48]. Alternatively, hydrogen peroxide may act as a reducing agent (Cu(II) + H_2_O_2_ → Cu(I) + OH^−^ + ^•^OOH) forming thus perhydroxyl radical ^•^OOH. We note, that mechanism in which copper (and iron) promote formation of hydroxyl radical via Fenton reaction is critically dependent on the concentration of dissolved dioxygen.

An analysis of electrophoregrams has shown that the studied complexes exhibited in the presence of hydrogen peroxide a concentration dependent effect on the conversion of supercoiled DNA (form I) to the nicked form II, followed by the linear form III. Activities of the complexes appear to be strongest at the concentration of 5 μM (Figure 11A–C, Line 6). Further increase in the concentration of the complexes resulted in the diminished nuclease activity seen in the gel as a gradual loss of a signal for linear DNA (form III) (Line 7–10) or the presence of a certain amount of intact circular form I. The slight smear seen in the line 6 (Figure 11A,B) is evidence of the DNA fragmentation as a consequence of stronger nuclease activity of the compounds.

Based on the electrophoretic profiles, the DNA cleavage ability of the complexes in the presence of hydrogen peroxide can be ordered as follows: **1** > **2** > **3**. In case of complexes **1** and **2** their interaction with DNA in DMSO is mediated by molecular structures of the complexes. Since the surface of DNA is negatively charged due to the phosphate ions present in the ribose-phosphate backbone and under *in vitro* conditions this negative charge is not compensated, the preferential interaction between positively charged complex **3** with negatively charged DNA in DMSO may play an important role in the proposed mechanism of action. It is reasonable to assume, that the higher destructive effect of complexes **1** and **2** on the DNA is a result of two possible mechanisms of action: i) the conversion of closed circular DNA (form I) to the nicked DNA (form II) caused by the copper complex itself and ii) damage of DNA by Reactive Oxygen Species (ROS) – products of the interaction of copper with hydrogen peroxide.

## 3. Experimental Section

### 3.1. Chemical Reagents

All the chemicals used were of reagent grade (Sigma-Aldrich, Milwaukee, WI, USA; Serva, Heidelberg, Germany; or Merck, Darmstadt, Germany) and used without further purification. The organic reagents were purchased from Sigma-Aldrich; their purity was checked by NMR Spectroscopy. All of the buffer solutions used in DNA experiments were prepared using purified water (Simplicity Ultrapure Water System, Millipore, Bedford, MA, USA).

### 3.2. Synthesis of the Complexes

#### 3.2.1. [Cu(H_2_O)(5-Cl-Sal)(Neo)] (**1**)

Copper(II) acetate monohydrate (0.100 g, 0.5 mmol) was dissolved in ethanol (15 mL) and a solution of neocuproine, (0.104 g, 0.5 mmol) in ethanol (15 mL) was added with continuous stirring and heating (60 °C). To this royal blue solution a clear solution of 5-chlorosalicylic acid (0.086 g, 0.5 mmol) in ethanol (15 mL) was added. After 15 min of stirring, 10 mL of water were added. The solution was stirred for 20 min at 60 °C, filtered and allowed to cool. From this solution, green crystals of **1** were obtained (yield 80%). Analysis found: C 54.71, H 3.83, N 5.96%; calculated: 54.79, H 3.72, N 6.08.

#### 3.2.2. [Cu(μ-Sal)(Neo)]_2_ (**2**)

Copper(II) acetate monohydrate (0.200 g, 1 mmol) was dissolved in ethanol (20 mL) and a solution of neocuproine (0.208 g, 1 mmol) in ethanol (15 mL) was added with continuous stirring and heating (60 °C). To this royal blue solution a clear solution of salicylic acid (0.138 g, 1 mmol) in ethanol (15 mL) was added. After 5 min of stirring, 40 mL of water were added. The solution was stirred for 20 min at 60 °C, filtered and allowed to cool. Slow evaporation of the filtrate at room temperature yielded crystals which under the microscope could be separated into two types. However only one of them was found to correspond to complex **2** (yield 35%). Crystals obtained together with crystals of complex **2** were not suitable for X-ray analysis, therefore the optimization of synthesis was necessary. The ratio of reagent was changed (copper(II) acetate monohydrate (0.200 g, 1 mmol), neocuproine (0.208 g, 1 mmol), salicylic acid (0.276 g, 2 mmol)) and a methanol-ethanol solution (in a ratio of 2:1) was used as a solvent (60 mL). The solution was stirred for 2 days at 60 °C, filtered and allowed to cool. After a few days small crystals of **2** were obtained (yield 65%). Analysis found: C 61.87, H 4.11, N 6.72%; calculated: 61.83, H 3.95, N 6.87.

#### 3.2.3. [Cu_2_(μ-5-Cl-Sal)(5-Cl-HSal)_2_(Neo)_2_]·EtOH (**3**)

Copper(II) acetate monohydrate (0.200 g, 1 mmol) was dissolved in ethanol (20 mL) and a solution of neocuproine (0.208 g, 1 mmol) in ethanol (15 mL) was added with continuous stirring and heating (60 °C). To this royal blue solution a clear solution of 5-chlorosalicylic acid (0.346 g, 2 mmol) in ethanol (15 mL) was added. After 5 min of stirring, 40 mL of water were added. The solution was stirred for 20 min at 60 °C, filtered, allowed to cool and left to stand at room temperature, giving crystals of **3** suitable for X-ray analysis (yield 72%). Analysis found: C 55.11, H 3.50, N 5.72%; calculated: 55.66, H 3.34, N 5.30.

### 3.3. Analysis and Physical Measurements

Infrared spectra in the region of 400 to 4000 cm^−1^ were recorded on a Nicolet 5700 FT-IR spectrometer (Thermo Scientific, Waltham, MA, USA). Spectra of the solid samples were obtained by the ATR technique at room temperature. Electronic spectra (9000–50,000 cm^−1^) of the powdered samples in Nujol mulls were recorded at room temperature (r.t.) on Specord 250 PLUS spectrophotometer (Analytik Jena AG, Jena, Germany). The contents of hydrogen, nitrogen and carbon in the samples were determined by elemental analysis using a commercial micro analyzer FLASH EA 1112 (ThermoFinnigan, San Jose, CA, USA).

### 3.4. X-ray Crystallography

The data were collected at 298.0(1) K on an Oxford Diffraction (Oxford, UK) Kappa geometry GEMINI R diffractometer equipped with Ruby CCD area detector using graphite monochromated MoKα radiation (*λ* = 0.71073 Å) at 50 kV and 40 mA. Crystal to detector distance was 53 mm. The diffraction intensities were collected and integrated and the Lorentz-polarization and FACE-absorption correction were performed with CrysAlis CCD RED software [49]. The crystal structures were solved by direct methods using SHELXS-2013 [50] and refined by the full-matrix least squares procedure with SHELXL-2014 [51]. Details of the X-ray diffraction experiment conditions and the crystallographic data for compounds **1**, **2** and **3** are given in Table 5.

**Table 5:** Crystal data and structure refinement parameters for compounds **1**, **2** and **3**.

	1	2	3
Chemical formula	C_21_H_17_O_4_N_2_CuCl	C_42_H_32_O_6_N_4_Cu_2_	C_51_H_41_O_10_N_4_Cu_2_Cl_3_
M_r_	460.37	815.82	1103.31
Cell setting, space group	Monoclinic, *C*2/_c_	Orthorhombic, *P*bcn	Orthorhombic, *P*2_1_2_1_2_1_
*T* (K)	298	298	298
*a* (Å)	22.8966 4)	20.579(3)	11.3988(2)
*b* (Å)	11.1471(2)	7.824(1)	13.7685(2)
*c* (Å)	15.7536(2)	21.432(2)	30.6145(4)
*α* (°)	90	90	90
*β* (°)	104.366(2)	90	90
*γ* (°)	90	90	90
*V* (Å^3^)	3895.1(2)	3451.0(7)	4804.8(2)
*Z*	8	4	4
μ (mm^−1^)	1.290	1.291	1.116
Crystal size (mm)	0.3174 × 0.2481 × 0.2118	0.2990 × 0.0511 × 0.0272	0.1294 × 0.1114 × 0.0300
*T_min_*, *T_max_*	0.688, 0.761	0.924, 0.965	0.866, 0.967
*S*	1.030	0.815	0.842
*R_1_*	0.0343	0.0423	0.0319
Data/restraints/parameters	3966/3/268	3515/0/24	9789/7/640
CCDC No.	1036514	1036515	1036416

ORTEP plots of the complexes **1**, **2** and **3** were drawn by DIAMOND [52]. The positions of the water H atoms obtained from a difference Fourier map were constrained to ideal water geometry and fixed in the final stages of refinement (O-H 0.85 or 0.95 Å). All other H atoms were included in calculated positions, with C-H distances ranging from 0.93 to 0.96 Å. They were refined in the riding-model approximation, with Uiso(H) = 1.2 Ueq (C) or 1.5 Ueq(C, O).

### 3.5. EPR Spectroscopy

The EPR spectra of frozen solutions (77 K) of copper complexes were measured using an EMX spectrometer (operating at X band, with 100-kHz field modulation) (Bruker, Karlsruhe, Germany) that was interfaced with a PC for data acquisition. The *g* factors were quoted with an uncertainty of 0.001 using an internal reference standard marker containing 1,1-diphenyl-2-picrylhydrazyl (DPPH) built into the EPR spectrometer. Cylindrical quartz sample tubes with 3.5 mm 0.d. (*ca.* 3.0 mm i.d.) were used for measurements.

### 3.6. DNA Cleavage Activity

#### 3.6.1. Purification of Plasmid DNA

Plasmid DNA pBSK+ was purified from *E. coli* strain DH5α using commercially available kit (Isolate II Plasmid Mini Kit, Bioline, Taunton, MA, USA). *E. coli* DH5α containing plasmid pBSK+ was grown in LB/Amp medium for 16 h at 37 °C under continual shaking (150 rpm, Heidolf Unimax 1010 incubating shaker, Schwabach, Germany). Plasmid DNA pBSK+ was purified from the cell pellet following the instruction in the isolation protocol. The phosphate buffer (pH 7.4) was used to elute the DNA. DNA concentration was measured on a NanoDrop ND-1000 spectrophotometer (Nanodrop Technologies, Wilmington, DE, USA). Absorbance ratios A260/A230 in the range 2.0–2.2 and A260/A280 ≥ 1.8 were determined for all DNA used and indicated a sufficient purity of DNA samples.

#### 3.6.2. DNA Cleavage by Copper Complexes

The DNA cleavage activity of the complexes was studied by agarose gel electrophoresis. The reaction contained 15 µM of pBSK + DNA in 50 mM sodium phosphate buffer (pH 7.4) and the solution of the copper complex in DMSO with the following concentrations 5; 10; 25; 40; 50 µM. Experiments were performed in the absence and presence of hydrogen peroxide (50 µM H_2_O_2_). The indicated concentrations are final concentrations in a volume of 20 µL. Along with these variants two positive controls with DMSO and H_2_O_2_ were prepared. Samples were incubated for 1 h at 37 °C and then mixed with the loading buffer (4 µL) containing 0.25% bromophenol blue in 30% glycerol. Samples were separated on the 0.8% agarose gel in TBE (89 mM Tris-borate acid, 2 mM EDTA, pH = 8.0) for about 1.5 h at 80 V. The 1 kb DNA ladder (Solis BioDyne, Tartu, Estonia) was used as a standard. Reference plasmid sample was linearized with EcoRI endonuclease (Fast digest EcoRI, Fermentas) and used as a control for double strand breaks. A gel was stained with 0.5 µg/mL of ethidium bromide and imaged under UV light (Kodak Gel Logic 200, Eastman Kodak Company, Rochester, NY, USA). Each experiment was performed in triplicate, under the same conditions.

## 4. Conclusions

Three novel coordination complexes have been prepared and characterized by means of X-ray crystallography, IR and EPR spectroscopy. The study of potential biological properties of prepared complexes has been also performed. Our preliminary study on the DNA cleavage activity has shown that the complexes under study behave as chemical nucleases in the presence of hydrogen peroxide with slight differences in the activity (**1** > **2** > **3**). The complexes are more efficient in DNA degradation process at the lowest tested concentration of complex compound (5 μM). The complexes **1** and **2** exhibited nuclease activity itself indicating the interaction of complexes with the DNA. As it has been shown previously [43], while derivatives of salicylic acids in the coordination environment of copper(II) complexes are responsible for radicals scavenging activity (predominantly towards superoxide radical anion), incorporation of chelating ligand 2,9-dimethyl-1,10-phenantroline into the copper(II) complexes significantly enhances their capability of binding to DNA via intercalation. Based on the presented results, it may concluded that the relative binding strength of the studied copper(II) complexes to DNA may be an important element of the mode of action of these three coordination compounds. The results suggest that the two methyl groups in the neocuproine molecule do not cause steric obstacles which impede the intercalation of the copper(II) complexes to DNA.

## Supplementary Materials

Crystallographic data (excluding structure factors) for the structures reported in this paper have been deposited with the Cambridge Crystallographic Data Centre as supplementary publication nos. CCDC-1036514 (**1**), CCDC-1036515 (**2**), and CCDC-1036516 (**3**). Copies of the data can be obtained free of charge on application to the CCDC, 12 Union Road, Cambridge CB2 1EZ, UK [fax: (internat.) +44 1223/336033; e-mail: deposit@ccdc.cam.ac.uk].
